# Changes in α-Dicarbonyl Compound Contents during Storage of Various Fruits and Juices

**DOI:** 10.3390/foods13101509

**Published:** 2024-05-13

**Authors:** Yang Yang, Xue-Yi Liu, Qian Zhao, Dan Wu, Jin-Tao Ren, Meng Ma, Pei-Yun Li, Jia-Cai Wu, Wen-Yun Gao, Heng Li

**Affiliations:** 1 College of Life Sciences, Northwest University, 229 North Taibai Road, Xi’an 710069, China; yangnova@xiyi.edu.cn (Y.Y.); liuxueyi@stumail.nwu.edu.cn (X.-Y.L.); z1502953450@163.com (Q.Z.); wudan0416d@126.com (D.W.); 18392535350@163.com (J.-T.R.); 19903765157@163.com (M.M.); l159753py@163.com (P.-Y.L.); 15929212135@163.com (J.-C.W.); gaowenyun@nwu.edu.cn (W.-Y.G.); 2 School of Pharmacy, Xi’an Medical University, 1 Xinwang Road, Xi’an 710021, China

**Keywords:** 3-deoxyglucosone (3-DG), glyoxal (GO), methylglyoxal (MGO), fruits, juices, storage

## Abstract

α-Dicarbonyl compounds (α-DCs) are commonly present in various foods. We conducted the investigation into concentration changes of α-DCs including 3-deoxyglucosone (3-DG), glyoxal (GO), and methylglyoxal (MGO) in fresh fruits and decapped commercial juices during storage at room temperature and 4 °C, as well as in homemade juices during storage at 4 °C. The studies indicate the presence of α-DCs in all samples. The initial contents of 3-DG in the commercial juices (6.74 to 65.61 μg/mL) are higher than those in the homemade ones (1.97 to 4.65 μg/mL) as well as fruits (1.58 to 3.33 μg/g). The initial concentrations of GO and MGO are normally less than 1 μg/mL in all samples. During storage, the α-DC levels in the fruits exhibit an initial increase followed by a subsequent decrease, whereas, in all juices, they tend to accumulate continuously over time. As expected, 4 °C storage reduces the increase rates of the α-DC concentrations in most samples. From the viewpoint of the α-DC contents, fruits and homemade juices should always be the first choice for daily intake of nutrients and commercial juices ought to be mostly avoided.

## 1. Introduction

3-Deoxyglucosone (3-DG), glyoxal (GO), and methylglyoxal (MGO) are common α-dicarbonyl compounds (α-DCs) found in various food products and are typically generated through non-enzymatic procedures during food processing and storage [1]. The production of α-DCs is largely impacted by the food ingredients, chemical environment, and processing and storage parameters [2,3]. The existence of these kinds of compounds in foods has received more and more attention in recent years, primarily due to the role they play in the formation of color, aroma, and flavor profiles in a number of foods [4]. Furthermore, they are active components that decorate the free amino or mercapto groups in the side chains of a protein covalently in the course of food processing and storage, leading to the formation of advanced glycation end products (AGEs) and cross-linking in various foods, which induce minus impact on the properties and stability of the proteins [5,6]. α-DCs are also found in the body fluids of human beings. Studies have shown that over-accumulation of these compounds in the body fluids triggers carbonyl stress and may severely damage the structure of a protein and have a bad effect on its function [7,8]. Further research has demonstrated that the generation and assembling of α-DCs in the body are implicated in many chronic diseases, such as diabetes and its complications [9], Alzheimer’s disease [10], cardiovascular disease [11], and cancer [12]. Therefore, conducting research on food α-DCs is imperative for ensuring food quality and safeguarding human health.

Fruits serve as a vital source of vitamins, minerals, antioxidants, and dietary fiber, which are essential for the human body’s well-being. The amounts of α-DCs in fruit products, such as fruit juices and dried fruits, have been extensively documented [13,14]. To regulate and minimize the levels of α-DCs in fruit products, researchers have examined the impact of processing and storage parameters on the variability of α-DCs in fruit products and established a multi-response kinetic model for the generation of α-DCs in fruit juices to investigate their formation mechanism during processing and storage [15,16]. Fresh fruit is more relevant to the everyday life of individuals than fruit products. It has been shown that green plants also contain a certain amount of α-DCs and their related AGEs [17]. However, to date, there are scarce reports on the determination of α-DCs in fruits and no studies have investigated the changes in α-DC contents during fruit storage. In addition, many families nowadays opt to prepare their own juices as a means of facilitating the intake of various nutrients from fruits while avoiding the ingestion of preservatives and/or sweeteners that are commonly added to commercial juices, but no studies have been conducted on how the contents of the α-DCs in such homemade juices alter during storage, either. 

In this study, we selected 6 fresh fruits, including apricot, black plum, nectarine, and mango, as the representatives of climacteric fruits, and sugar orange and red grape as the representatives of non-climacteric fruits, to analyze their contents of α-DCs such as 3-DG, GO, and MGO, respectively, and alterations of their levels during storage at 4 °C and room temperature (rt). Meanwhile, we also determined the variations of α-DC contents in 6 commercial juices stored at rt and 4 °C, as well as in 6 homemade juices stored at 4 °C. In this article, we disclose all the experimental results.

## 2. Materials and Methods

### 2.1. Chemicals and Reagents

Analytical-grade 3-DG was obtained from Toronto Research Chemicals (Toronto, ON, Canada), and GO (40% in water), MGO (32% in water), and 4-nitro-1,2-phenylenediamine (NPDA) were bought from Fluka (Shanghai, China). The 100 mM stock solutions of the three α-DCs were prepared in Millipore water, and NPDA (20.0 mM) was dissolved in methanol. All these solutions were kept at 4 °C until use. HPLC-methanol was from Sigma-Aldrich (Beijing, China). A Milli-Q water purification system (Milli-Q CLX 7000, Merck, Shanghai, China) was used to produce water of high purity. All other chemicals were of analytical purity and were used directly without further purification.

### 2.2. Instrumentation

HPLC analyses of the α-DCs were carried out on an Agilent 1260 HPLC system (Agilent Technologies, Shanghai, China) coupled with a double-beam UV detector, an auto-sampler, and a Shim-pack VP-ODS column (250 × 4.6 mm, 5 μm, Shimadzu, Beijing, China). HR-MS determination was performed on a Thermo Fisher LTQ XL apparatus (Thermofisher, Shanghai, China).

### 2.3. Fruits and Juices

Apricots (*Prunus armeniaca* L. cv. ‘Golden Sun’), black plums (*Prunus salicina* Lindl. cv. ‘Black amber’), nectarines (*Prunus persica* var. *nectarina* Maxim. cv. ‘Zhongnongjinhui’), and red grape (*Vitis vinifera* L. cv. ‘Hongti’) were harvested at the physiological mature stage from ‘Shaanxi Fruit Industry Group Co., LTD.’ orchards in Xi’an, China. Mangoes (*Mangifera indica* L. cv. ‘Tainong No.1’) and sugar oranges (*Citrus reticulata* Blanco. cv. ‘Shatangju’) were cultivated by ‘The Agricultural Cooperative of Qingfeng’ in Zhanjiang, China. Fruits were immediately transported to the laboratory in Xi’an through express delivery after harvesting. Each fruit was carefully selected to ensure uniformity in terms of color, size, and ripeness and was free from external damage and insect spots. For determination, each sample was divided into two groups. One group was stored in semi-open cartons at rt (20–25 °C) for 8–10 d, while the other was refrigerated at 4 ± 1 °C for 8–13 d. The relative humidity and temperature of the storage room and refrigerator were documented with EBI 20-TH1 humidity/temperature recorders (Ebro, Beijing, China). The average temperatures of the storage room and refrigerator were 22 ± 2.5 °C and 4.0 ± 0.6 °C and relative humidity were 66.2 ± 8.1% and 78.9 ± 6.3%, respectively. An exception was that mangoes were only kept at rt, since at 4 °C they could be cold-injured. From each group, 3 intact fruits were randomly opted daily for measurement until the end of storage. For red grape, 20 fruits were collected by chance from different clusters every day. Before being sliced, cored, and homogenized with a handheld kitchen blender on an ice bath, mangoes, oranges, and red grape were separately peeled, while the remaining fruits were washed with tap water and dried off, respectively. 

Six pure fruit juices (diluted from juice concentrate without extra sugar addition), including peach, orange, mango, pineapple, apple, and grape juices, were purchased from a universal fruit processing company in China, from where they were yielded. Two 20 mL aliquots of each juice were transferred separately in two screw-capped Eppendorf tubes, stored respectively at rt and at 4 °C for 7 days, and measured daily without any pretreatment. The homemade juices were made in the lab according to the traditional route of homemade raw juice preparation without adding any food additives. Briefly, peaches (*Prunus persica* (L.) Batsch. cv. ‘Okubao’), apples (*Malus pumila* Mill. cv. ‘Red Fuji’), and grape (*Vitis vinifera* L. cv. ‘Hongti’) from orchards in Xi’an and sugar oranges (*Citrus reticulata* Blanco. cv. ‘Shatangju’), mangoes (*Mangifera indica* L. cv. ‘Tainong No.1’), and pineapples (*Ananas comosus* (L.) Merr. cv. ‘Smooth Cayenne’) from orchards in Zhejiang were delivered to the laboratory via courier immediately after picking. Intact fruits were selected and washed with tap water, peeled, and squeezed separately with a commercial squeezer (Midea 101B, Zhengjiang, China). A 20 mL aliquot of each juice was transferred into a screw-capped Eppendorf tube and was measured immediately after juicing and after storage at 4 °C for 4–72 h. The juices were centrifuged separately at 10,950× *g*, 4 °C for 20 min before the supernatants were used for determination.

### 2.4. Extraction of the α-DCs from the Homogenate of the Fruits

The optimization of the fruit sample pretreatment process is detailed in Part I of the Supplementary Materials (SM). From the homogenate of each fruit obtained in Section 2.3, 10 g of a high-water content fruit (black plum, apricot, nectarine, sugar orange, or red grape) or 2.5 g of a low-water content fruit (mango) were weighed. Then, 5 mL of distilled water was added to each of the former homogenates and 10 mL was added to the latter for extraction. The homogenate of red grape was not supplemented with additional water as the homogenization process resulted in very little solid matter remaining. In the subsequent step, each mixture was vortexed individually at a speed of 2800 rpm for 5 min prior to being centrifuged at 10,950× *g* and 4 °C for a duration of 20 min. The generated supernatants were transferred into separate 25 mL volumetric flasks and the process was repeated once more. The resulting supernatants were then combined with their corresponding ones, and the volumes were adjusted with distilled water before being stored at 4 °C for subsequent derivatization.

### 2.5. Derivatization of the α-DCs in Various Fruit Extracts and Different Juices

Derivatization of the α-DCs in various fruit extracts and different juices with NPDA was carried out according to the published method [18]. A 670 μL portion of each of the fruit extracts (as in Part 2.4) or each of the juices in a 2 mL Eppendorf vial was added with 250 μL NPDA solution (20 mM) and 80 μL of Millipore water, and the mixture was vortexed on a mixer. After mixing well, the pH of the assay solution was adjusted to 9.0. Then, the mixture was reacted in the dark for 2 h at rt. Finally, the solution was used for HPLC analysis after it had been filtered through a membrane of 0.22 μm.

### 2.6. HPLC-UV Determination of NPDA Derivatives of the α-DCs 

The HPLC profiles of NPDA and it derivatized 3-DG, GO, and MGO and the validation of the HPLC-UV process are presented in Parts II and III in the SM. NPDA-labeled α-DCs were measured utilizing the published methods with minor modifications [18,19]. A mixture of methanol-water (65:35, *v*/*v*) was used as the mobile phase after filtration through a membrane of 0.22 μm, and the determinations were performed in an isocratic mode at ambient temperature (injection volume: 10 μL; flow rate: 0.7 mL/min; UV wavelength: 265 nm). Each sample was injected parallelly in triplicate. The LC-ESI-HRMS characterization and the retention times are given in Table SM2. One HPLC run could be done within 12 min.

### 2.7. Statistical Methods

One-way ANOVA was performed to evaluate the effect of storage on the concentrations of the three α-DCs in fruits and juices. The Statistical Package for the Social Sciences (SPSS 17.0) was utilized for calculating the statistical significance of the discrepancies between the averages through the Duncan test. *p* < 0.05 was set to be of statistical significance for the results. 

## 3. Results and Discussion

### 3.1. Weight Loss of Fruits during Storage

Changes in the weight of fruits during storage under two different conditions are given in Supplementary Table S1. The data show that the weight loss of the fruits during 4 °C storage was not significant until 4 days, except in red grape.

### 3.2. Changes in the Concentrations of the Three α-DCs in Fruits during Storage

#### 3.2.1. Alteration of 3-DG Contents

**Figure 1:**
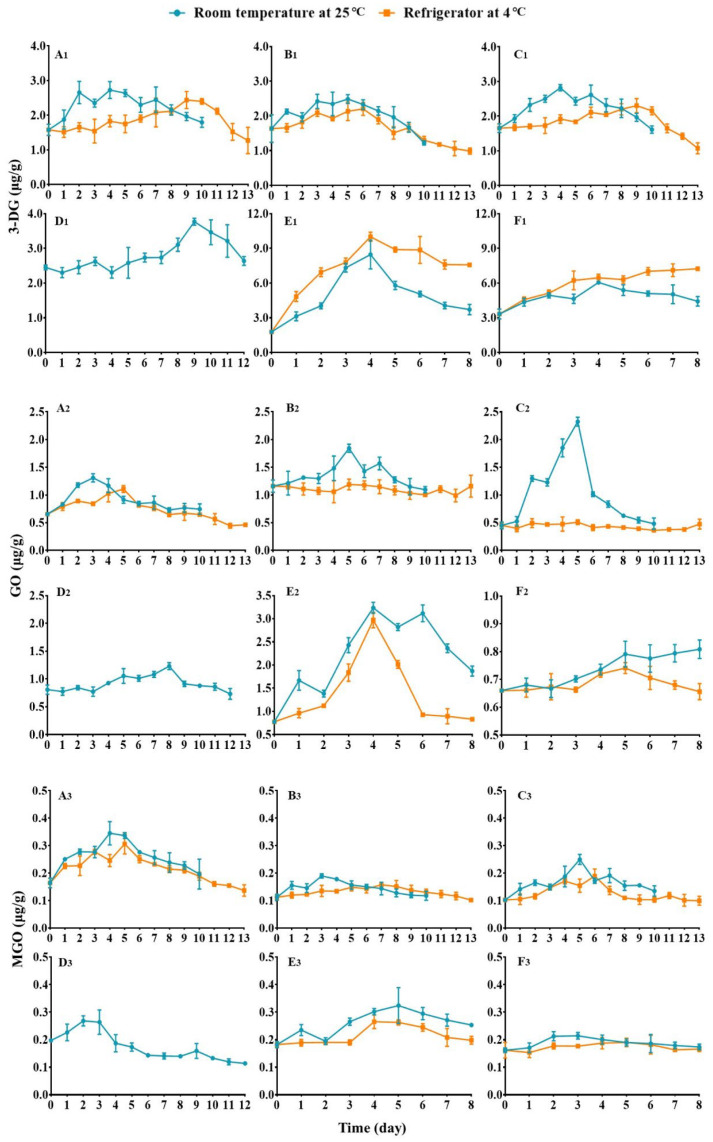
The changes of α-DC contents in different fruits stored at room temperature (blue line) and 4 °C (orange line) for 8–13 days. (**A_1_**–**F_1_**): the changes of 3-DG contents in apricot (**A_1_**), black plum (**B_1_**), nectarine (**C_1_**), mango (**D_1_**), sugar orange (**E_1_**), and red grape (**F_1_**) (*n* = 3). (**A_2_**–**F_2_**): the changes of GO contents in apricot (**A_2_**), black plum (**B_2_**), nectarine (**C_2_**), mango (**D_2_**), sugar orange (**E_2_**), and red grape (**F_2_**) (*n* = 3). (**A_3_**–**F_3_**): the change of MGO content in apricot (**A_3_**), plum (**B_3_**), nectarine (**C_3_**), mango (**D_3_**), sugar orange (**E_3_**) and red grape (**F_3_**) (*n* = 3).

The initial contents of 3-DG (zero-day storage) in the selected fruits are listed in Table 1, and their contents at different storage periods are given in Figure 1A_1_–F_1_ (the original data are given in Supplementary Tables S5–S15). In general, it can be observed that: (i) the 3-DG contents of all fruits exhibit an initial increase followed by a subsequent decrease during storage, regardless of fruit type and storage temperature. However, red grape stored at 4 °C display a continuous rise in 3-DG content; (ii) the daily increase rates of 3-DG ((daily determined value—initial value)/initial value) in the climacteric fruits, including apricot (A1), black plum (B1), and nectarine (C1), are higher when they are stored at rt (blue line) than when stored at 4 °C within the first 8 days (orange line); in contrast, in the non-climacteric fruits such as sugar orange (E1) and red grape (F1), the daily increase rates are lower when they are stored at rt than when stored at 4 °C during the whole storage period (8 days), exhibiting that 3-DG content change is fruit type- and storage temperature-dependent. Having further reviewed the data, we realize that some fruits display distinct peak times and daily increase rates of 3-DG contents from the others. When kept at rt, the compound in all the fruits except for mango attains its peak concentrations after 4–5 days (peak time), while mango takes double the time to complete this process. The maximum increase rates of 3-DG in five fruits, namely apricot, black plum, nectarine, mango, and grape, are almost at the same level (50–80%), but in orange, the value is about 370%, indicating that 3-DG content in the fruit grows nearly 3 times at its peak (Table 1). When kept at 4 °C, different phenomena are observed. The peak times of the compound in apricot, nectarine, and grape are doubled, while in black plum and orange, the peak times remain almost unchanged. Regarding the maximum increase rates, it is noteworthy that in climacteric fruits such as apricot, black plum, and nectarine, the values range from approximately 35% to 55%, which are lower than those obtained for corresponding fruits stored at room temperature. However, non-climacteric fruits such as orange and red grape exhibit contrasting results; their maximum increase rates of 3-DG are around 460% and 120%, respectively—both rates are significantly higher than those acquired for the two fruits stored at room temperature (Figure 1 and Table 1). These data disclose the different responses of 3-DG contents in the climacteric and non-climacteric fruits to the storage conditions, yet the underlying factor driving this phenomenon remains unclear.

The factors affecting 3-DG content inside fruit include the rates of its production from soluble sugars and its transformation to a series of downstream products. On the one hand, glucose and fructose, the major soluble sugars, can undergo dehydration to yield 3-DG [13,20]. On the other hand, the degradation of 3-DG leads to the formation of 5-HMF, 3,4-DGE, and MGO [15,21]. In addition, some 3-DG metabolizing enzymes present in fruit transform 3-DG to 3-deoxyfructose [22,23,24]. Therefore, the initial increase in 3-DG content during storage may be attributed to its faster formation rate compared to consumption; while with its continuous accumulation, various protective mechanisms inside the fruits may inhibit its production and cause a shift in conversion, resulting in fluctuations of 3-DG content from increase to decrease. 

As previously mentioned, the climacteric fruits (apricot, plum, and nectarine) exhibit a contrasting storage temperature response to non-climacteric fruits (orange and grape) in terms of their daily increase rates of 3-DG contents. The former displays greater increases in 3-DG content (1–8 days) when stored at rt, while the latter shows smaller increases under the same conditions (Figure 1 and Table 1). The phenomena could be due to:

(i) The climacteric fruit undergoes a distinct post-ripening process, during which its respiratory rate suddenly speeds up, along with a rapid increase in soluble sugars [25,26], resulting in the acceleration of 3-DG production [27]. At 4 °C, however, the post-ripening process is inhibited to some extent, retarding the accumulation of soluble sugars in fruit and accordingly slowing down the increase rate of 3-DG contents;

(ii) The non-climacteric fruit lacks an obvious post-ripening process [25,26], so the storage temperature exerts a little influence on its soluble sugars and hence on the production of 3-DG. The degradation of the compound, however, proceeds regularly at ambient temperature, but is suppressed at 4 °C; for instance, the conversion of 3-DG to 5-HMF through dehydration has been reported to occur at rt but not at low temperatures [28,29]. The trait of this kind of fruit causes the consumption rates of 3-DG contents to be higher when stored at rt than when kept at 4 °C.

#### 3.2.2. Alteration of GO Contents

The initial GO contents in the selected fruits and their variations during different storage periods are illustrated in Figure 1 and Table 1, respectively (the original data are given in Supplementary Tables S5–S15). As depicted in Figure 1A_2_–F_2_, the fluctuations of GO levels in diverse fruits generally exhibit an ascending-descending pattern during storage at either 4 °C or rt, which is analogous to that of 3-DG. The exceptions are black plum (B2) and nectarine (C2), which indicate no significant variation in GO contents at 4 °C, and red grape (F2), which shows a continuous rise in GO content at rt. As for the daily increase rates of the compound, the climacteric fruits apricot (A2), black plum, and nectarine exhibit a similar trend to 3-DG in that their GO increase rates are greater when stored at rt compared to 4 °C. Conversely, non-climacteric fruits such as orange (E2) and grape demonstrate an opposing result to 3-DG, whereby their daily GO increase rates are higher when stored at rt rather than at 4 °C. From the data listed in Table 1, we find that the initial contents of GO are generally much lower than those of 3-DG in the corresponding fruit. The peak times of GO in the fruits stored at rt closely match those of 3-DG in the relevant fruit, except for red grape, since it does not furnish a peak value; whereas, in the fruits stored at 4 °C, the situation differs significantly: except that the peak time of GO in orange remains consistent with that of 3-DG within the same fruit, black plum and nectarine produce no clear peak value, and the peak time of GO in apricot is almost doubled compared to at rt and is nearly only half of the peak time of 3-DG in the same fruit stored at 4 °C. As for the maximum increase rates of GO, apricot, black plum, mango, and orange stored at rt and apricot stored at 4 °C show comparable data to those of 3-DG in the homologous fruits kept under the same conditions. However, nectarine and red grape behave quite differently: the datum in nectarine stored at rt is bigger than 400%, displaying that the peak content of GO is about 4 times as high as the initial one; while in red grape stored at both temperatures, only around 22% and 12% maximum increase rates in GO are observed, which are much smaller than the ones determined for 3-DG in the same fruit stored separately at rt and 4 °C. 

According to the published data, the precursors of GO are glucose and fructose, just like those of 3-DG; the difference is that GO formation pathways involve oxidation steps [20,30]. Hence, the rate of GO production can be influenced by the oxygen level and antioxidant content in fruits. Moreover, polyphenolic compounds abundant in fruits may potentially entrap GO [31,32,33]. According to these discussions, the alteration of GO contents in the fruits during storage can be partially interpreted as follows:

(i) When stored in semi-open cartons at rt, fruits receive ample oxygen supply. Therefore, the factors influencing GO contents are identical to those of 3-DG. Consequently, it can be observed that the variation tendency and peak times of GO contents are consistent with those of 3-DG in relevant fruits. When refrigerated at 4 °C, two adverse factors inhibit the formation of GO in the climacteric fruits: low temperatures decrease the production of glucose and fructose and insufficient oxygen supply reduces the rate of the oxidation reactions. Therefore, almost no change in the GO contents in black plum and nectarine is observed. For the non-climacteric fruits orange and red grape, only the oxygen level may influence the GO formation, as the low temperature has no substantial effect on their production of soluble sugars [34]. The data depicted in Figure 1E_2_,F_2_ show that the deficient oxygen provision within the refrigerator seems to exert no obvious effect on GO contents in the two fruits because the levels of GO vary in parallel with those of 3-DG in the corresponding fruits; 

(ii) As described above, the daily increase rates of GO in all the six fruits show identical tendencies, with higher values observed at rt compared to those stored at 4 °C. For the three climacteric fruits, these tendencies are analogous to those of 3-DG in the relevant fruits and can be explained accordingly. For the two non-climacteric fruits, their tendencies are opposite to those of 3-DG in the relevant fruits (Figure 1E_1_,F_1_). This may be due to the fact that both the sufficient oxygen supply and the relatively high temperature are conducive to oxidation reactions when stored at high temperatures, promoting the formation of GO in the fruit; when stored in a 4 °C refrigerator, however, the formation of GO is suppressed because of the low temperature and low oxygen level. 

#### 3.2.3. Alteration of MGO Contents

The initial contents of MGO in the fruits and its contents at different storage periods are given in Figure 1 and Table 1, respectively (the original data are given in Supplementary Tables S5–S15). As shown in Figure 1A_3_–F_3_, the changes in MGO content of distinct fruits during storage at either 4 °C or rt follow the same tendency as that of GO, with an initial increase followed by a subsequent decrease. The daily increase rates of the compound in the six fruits exhibit a similar trend to that of GO. Specifically, the data for MGO in these fruits are greater when stored at rt compared to at 4 °C; the discrepancy is that the daily increase rates of MGO in all the fruits are small and no significant promotions in the levels of MGO contents were detectable during storage. These phenomena are opposite to those observed for GO contents in nectarines stored at rt (blue line in Figure 1C_2_) or oranges stored at both rt and 4 °C (Figure 1E_2_). The data listed in Table 1 display that the initial MGO contents in these fruits are much lower than those of GO in the relevant fruits. The peak times of the compound at rt correspond closely to those of GO in the relevant fruits, yet mango exhibits a unique pattern, with MGO content reaching its peak within two days, which contrasts sharply with the peak times observed for 3-DG and GO in the same fruit.

Compared with the sources of 3-DG and GO, we notice that MGO may originate from more precursors. Actually, it is a side-product of glycolysis, lipid peroxidation, oxidative degradation of glucose and glycated proteins, and photosynthesis [35]. Despite its multiple origins, its initial contents in all the selected fruits are as low as 10–25% of initial GO contents, with a maximum increase rate during rt storage of up to 145% (nectarine) (Table 1). This could be because of the mutagenic and genotoxic effects of MGO at relatively high concentrations. In response, plants have developed glyoxalase and non-glyoxalase systems that act synergistically to remove excess MGO [36]. In addition, diverse polyphenols present in fruits are also able to trap MGO [31,32,33].

### 3.3. Changes in the Concentrations of the Three α-DCs in Commercial Juices during Storage

#### 3.3.1. Alteration of 3-DG Contents

**Figure 2:**
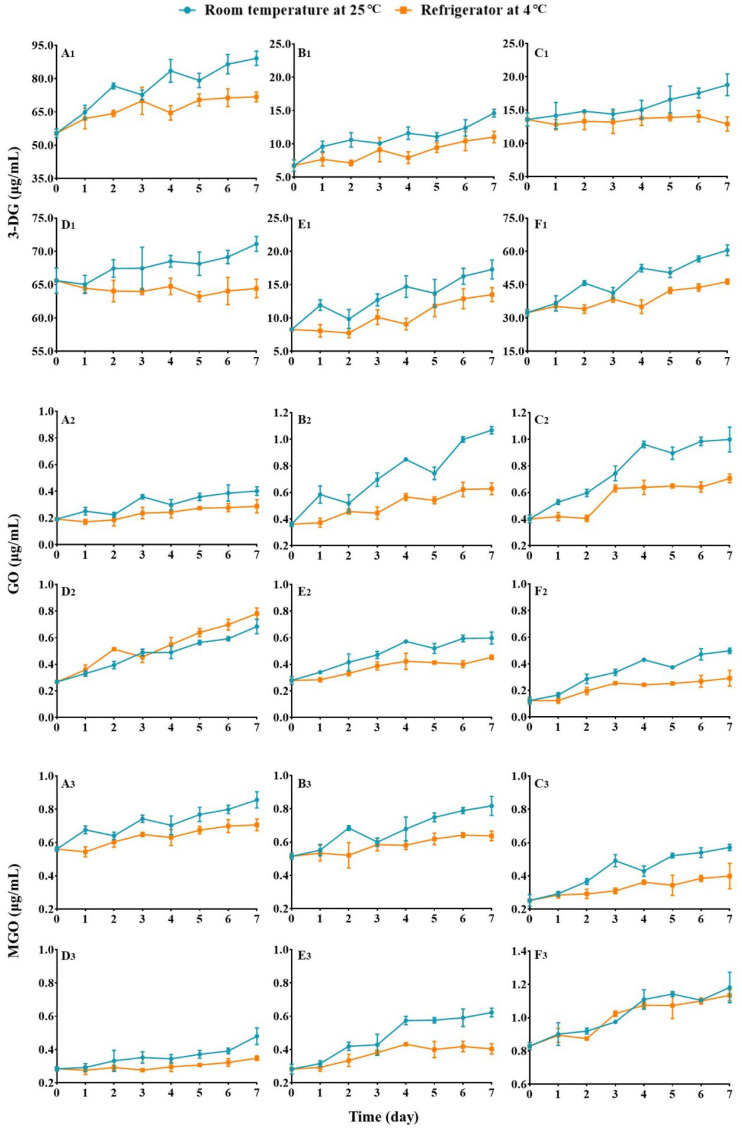
The changes of α-DCs contents in different juices stored at rt (blue line) and 4 °C (orange line) for 7 days. (**A_1_**–**F_1_**): the changes of 3-DG contents in apple juice (**A_1_**), mango juice (**B_1_**), orange juice (**C_1_**), grape juice (**D_1_**), pineapple juice (**E_1_**), and peach juice (**F_1_**) (*n* = 3); (**A_2_**–**F_2_**): the changes of GO contents in apple juice (**A_2_**), mango juice (**B_2_**), orange juice (**C_2_**), grape juice (**D_2_**), pineapple juice (**E_2_**), and peach juice (**F_2_**) (*n* = 3); (**A_3_**–**F_3_**): the changes of MGO contents in apple juice (**A_3_**), mango juice (**B_3_**), orange juice (**C_3_**), grape juice (**D_3_**), pineapple juice (**E_3_**), and peach juice (**F_3_**) (*n* = 3).

The concentrations of 3-DG in six commercially available juices at the outset and during various storage periods are presented in Figure 2 and Table 2, respectively (the original data are given in Supplementary Tables S16–S27). The direct observations suggest that the concentrations of 3-DG in most juices stored at both temperatures increase over time, with the exception of orange (C1) and grape (D1) juices, which exhibit relatively stable 3-DG levels when stored at 4 °C. The phenomena are entirely distinct from those witnessed in fruit storage in which (i) the alterations of 3-DG contents always follow a raise and then decrease pattern, no matter which storage temperature it is under; (ii) the increases of 3-DG content appear to be fluctuating rather than following a linear upward trend; (iii) the daily increase rates of 3-DG in the juices stored at rt are generally significantly larger than those in the relevant juices stored at 4 °C. The data given in Table 2 show that: (i) the initial concentrations of 3-DG vary significantly among these juices; for instance, the 3-DG level in grape juice is almost nine times higher than that in mango juice; (ii) 3-DG concentrations in the juices are much higher in general than those in the fruits. Among all the juices, mango juice has the lowest concentration of 3-DG, but it is still 2.7 times higher than that in mango fruit; (iii) different juices exhibit distinct maximum increase rates; for example, mango and pineapple juices show the maximum increase rates of bigger than 100%, whereas grape juice only displays a value of less than 10%. All these results imply that: (i) the various protective mechanisms that can restrict the concentration of 3-DG in a relatively low level inside fruit have been partially or severely impaired, resulting in a continuous rise of 3-DG contents in the juices; (ii) different juices possess discrete ways of action in limiting the growth of 3-DG concentrations. Furthermore, the data indicate that refrigeration of the juices inhibits the accumulation of 3-DG by suppressing dehydration reactions of soluble sugars such as glucose and fructose.

#### 3.3.2. Alteration of GO Contents

The concentrations of GO in the six commercial juices at the initial stage and during storage over time are illustrated in Figure 2 and Table 2, respectively (the original data are given in Supplementary Tables S16–S27). As shown in Figure 2A_2_–F_2_, under the two different storage temperatures, the GO concentrations in the juices gradually increase over time, resembling the concentration change trends of 3-DG in the juices, but contrasting with the change of GO contents in the fruits. Additionally, the daily increase rates of GO follow a similar trend to that of 3-DG in the juices, whereby storage at rt generally results in faster concentration increases of GO in the juices. However, grape juice displays an inverse tendency where the daily growth rates of GO are greater at 4 °C than at rt, except for on the third day. A rational explanation for this phenomenon has yet to be determined. The data presented in Table 2 demonstrate unexpectedly low initial concentrations of GO in these juices, which are only half of those found in the corresponding fruits mango, orange, and grape (Table 1). The maximum increase rates of GO, however, are significantly larger at both storage temperatures than those of 3-DG, and the data for grape and peach kept at rt are even 18 times and almost 4 times as great as those of 3-DG in the same juices stored at rt, respectively. 

As we have discussed, the level of GO in juice should also depend on two aspects, conversion from glucose and fructose, which needs the help of oxygen, and consumption by antioxidants and trapping agents [37,38]. On the one hand, commercial juice is normally kept in sealed containers inside which the oxygen level is low, leading to the formation of GO being fundamentally inhibited; on the other hand, the existing GO could be consumed by reduction and trapping. The synergistic action of these two factors results in a consistently low concentration of GO in the juice. After being uncapped, the exposure of juices to air promotes GO generation, but no extra antioxidants and polyphenols are supplemented. Moreover, various protective mechanisms present in fruit are more or less damaged during the juicing process. Consequently, GO concentrations in the juices start to increase quickly. Comparable to that of 3-DG, low storage temperature retards the increased rates of GO in the juices as well, since at 4 °C the oxidation reactions, which are critical in the formation of the compound, are also suppressed, but the situation in grape juice (D2) is an exception, which deserves more investigation.

#### 3.3.3. Alteration of MGO Contents

The initial concentrations of MGO in the commercial juices and their concentrations at different storage days are presented in Figure 2 and Table 2, respectively (the original data are given in Supplementary Tables S16–S27). It can be seen from Figure 2A_3_–F_3_ that the alteration tendencies of MGO concentrations in the juices stored at rt and 4 °C follow those of 3-DG and GO in the identical matrix but differ from that of MGO in fruits stored under the same conditions. In terms of the daily increase rates of MGO in the juices, almost the same phenomenon as those that occurred in 3-DG and GO was observed, with the exception of peach juice (F3), which shows comparable growth rates of MGO concentrations in both temperature conditions. From the data depicted in Table 2, it is evident that the initial concentrations of MGO in these juices are generally high. For example, in mango, orange, and grape juices, the data are greater than those found in the relevant fruits (Table 1). This result is inverse to the observation that the initial contents of GO in the juices are much lower than those determined in the fruits. 

We have previously discussed the factors contributing to the persistent elevation of 3-DG and GO levels in juices stored at both rt and 4 °C, which can also be applied to elucidate the changes in MGO concentrations under identical conditions. Its higher initial concentrations in the juices might be because MGO is a multi-origin metabolite that could be produced by several non-enzymatic routes and removed by enzymatic and non-enzymatic pathways [31,32,36]. During juice processing, the enzymatic detoxification mechanism that limits MGO levels in fruit is crippled; thus, the MGO formation rate exceeds its removal rate, resulting in a concentration increase during storage. The reason that the initial contents of MGO in the juices are generally higher than those of GO could be owing to the fact that, under a sealed state, the generation of GO is inhibited to some extent, because of low oxygen supply and removal by trapping reagents, while the formation of MGO appears unaffected.

Recent studies revealed that during long-term storage of capped commercial juices at distinct temperatures, the levels of 3-DG, GO, MGO, and other dicarbonyl compounds show a storage period and temperature-dependent increase [16,28,39]. This observation is somewhat identical to what we have surveyed in the decapped juices in this study. 

### 3.4. Changes in the Concentrations of the Three α-DCs in the Homemade Juices during 4 °C Storage 

#### 3.4.1. Alteration of 3-DG Contents

**Figure 3:**
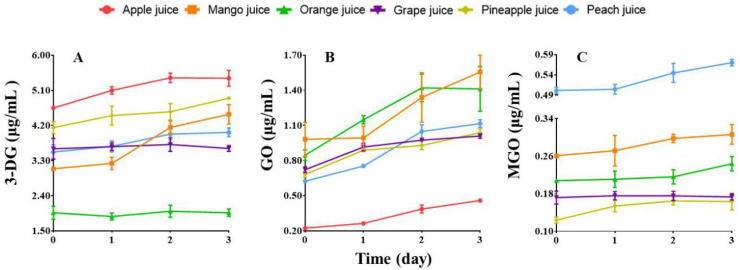
The changes of 3-DG (**A**), GO (**B**), and MGO (**C**) concentrations in homemade juices stored at 4 °C for 3 days (*n* = 3).

The initial concentrations of 3-DG in 6 homemade juices and their concentrations at different storage days are given in Figure 3 and Table 3, respectively (the original data are given in Supplementary Tables S28–S33). Due to the absence of protective reagents such as preservatives and antioxidants, these juices were only stored at 4 °C for three days. From Figure 3A, we can see that during storage, the 3-DG contents in apple, mango, pineapple, and peach juices only show a mild elevation, whereas in grape and orange juices, there is almost no increase in 3-DG concentration. These results are quite similar to those observed for the changes of 3-DG contents in the commercial juices stored at 4 °C. The data in Table 3 indicate the initial 3-DG contents in homemade juices are much lower than those found in relevant commercial ones, for example, in grape, apple, and peach juices, the 3-DG contents are only around 5–10% of those of corresponding commercial samples and are little higher than those in the fruits of mango, orange, and grape (Table 1). The maximum increase rates of homemade juices are generally smaller than those of commercial ones when stored at 4 °C. However, only homemade mango juice exhibits the opposite behavior with a higher rate than the commercial one. An interesting observation is that the 3-DG contents in both orange and grape juices, whether commercial or homemade, remain relatively stable during storage at 4 °C, which seems to deserve more investigation.

As we have demonstrated, the 3-DG contents in homemade juices are much lower than those found in commercial ones. Reasons for this could be: (i) The freshly squeezed homemade juices are instantly obtained from fresh fruits without undergoing lengthy processing procedures, thereby minimizing the extent of damage to the protective mechanisms in the fruits and significantly inhibiting the formation of 3-DG; (ii) No extra sweetener was added to the homemade juices. These factors may also account for the relatively lower increase rates of 3-DG levels observed in homemade juices compared to their commercial counterparts.

#### 3.4.2. Alteration of GO Contents

The initial concentrations of GO in 6 homemade juices and their concentrations at different storage days are also depicted in Figure 3 and Table 3, respectively (the original data are given in Supplementary Tables S28–S33). As shown in Figure 3B, the GO concentrations in the juices rise over the storage time, resembling the change trends of 3-DG but at quicker rates. Additionally, the increase rates of GO in these kinds of juices are typically greater than those of GO in relevant commercial samples when stored at 4 °C (Figure 2A_2_–F_2_). The data listed in Table 3 indicate that the initial concentrations of GO in the juices are much lower compared to the 3-DG contents in the same juice but are higher than those of GO in the corresponding commercial samples. Further comparison displays that the initial GO contents in mango, orange, and grape juices are basically at the same levels as those in the corresponding fruits (Table 1). 

We have discussed above the factors influencing the initial contents of GO and its variations during storage in commercial juices; these factors could affect the same GO in homemade ones. Due to the open atmosphere and lack of antioxidant supplementation during preparation, homemade juices exhibit higher initial concentrations of GO compared to their commercial counterparts. Furthermore, levels of GO in homemade juices increase rapidly during storage. 

#### 3.4.3. Alteration of MGO Contents

The initial concentrations of MGO in 6 homemade juices and their levels at different storage days are also presented in Figure 3 and listed in Table 3, respectively (the original data are given in Supplementary Tables S28–S33). The data in Figure 3C show that, during storage, the MGO contents in these juices rise at smaller increase rates, similar to what we have observed with 3-DG. Unexpectedly, no MGO is detectable in apple juice, while in the commercial one, MGO is present at a not very low level. The data in Table 3 indicate the initial contents of MGO in these homemade juices are generally lower than those of GO in the relevant homemade ones, which is contrary to the trend observed in commercial juices where the initial levels of MGO are typically higher than those of GO (Table 2). The reason for this could be that homemade juices contain factors that promote the production of GO, such as oxygen supply and the absence of additional protective reagents, while lacking factors that enhance the formation of MGO, such as added sugar. The data also display that the contents of MGO in these homemade juices are also apparently smaller than those in the corresponding commercial ones. This could be due to the fact that there is no extra supply of sugar in the homemade juices, either. Further comparison reveals that the initial MGO concentrations in mango, orange, and grape juices are at comparable levels to those in the corresponding fruits (Table 1).

## 4. Conclusions

In this study, we investigated for the first time changes in the contents of the three α-DCs including 3-DG, GO, and MGO in fresh fruits, uncapped commercial juices, and homemade juices during storage at different temperatures. Based on the aforementioned data and discussions, we recommend prioritizing fruit as a dietary choice, not only due to its low α-DCs concentrations but also because of its abundant supply of nutrients. Fresh homemade juice is a suitable option for both elderly individuals and infants due to its fruit-like characteristics, with the exception of dietary fiber. Moreover, if juices require storage, they should be tightly sealed and refrigerated for the shortest possible duration to preserve their α-DCs contents.

## Figures and Tables

**Table 1:** Initial values and alterations of the contents of the three α-DCs in different fruits stored at room temperature and 4 °C, respectively, for 8–13 days.

	Climacteric Fruits				Non-Climacteric Fruits	
3-DG	Apricot	Plum	Nectarine	Mango	Orange	Grape
Initial value (μg/g)	1.58	1.64	1.65	2.45	1.78	3.33
Peak value at rt (μg/g)	2.73	2.49	2.82	3.77	8.46	6.06
Peak time at rt (day)	4	5	4	9	4	4
Maximum increase rate at rt (%) ^a^	72.61	51.99	70.53	53.82	374.86	82.04
Peak value at 4 °C (μg/g)	2.44	2.20	2.30	NA ^b^	10.00	7.23
Peak time at 4 °C (day)	9	6	9	NA	4	NO ^c^
Maximum increase rate at 4 °C (%) ^a^	54.35	34.61	39.28	NA	461.32	117.36
GO						
Initial value (μg/g)	0.65	1.16	0.45	0.81	0.77	0.66
Peak value at rt (μg/g)	1.31	1.84	2.32	1.23	3.23	0.81
Peak time at rt (day)	3	5	5	8	4	NO ^c^
Maximum increase rate at rt (%) ^a^	100.39	58.94	414.93	52.44	318.90	22.55
Peak value at 4 °C (μg/g)	1.11	ND ^d^	ND	NA	2.97	0.74
Peak time at 4 °C (day)	5	ND	ND	NA	4	5
Maximum increase rate at 4 °C (%) ^a^	69.48	ND	ND	NA	285.15	12.32
MGO						
Initial value (μg/g)	0.16	0.11	0.10	0.20	0.18	0.16
Peak value at rt (μg/g)	0.34	0.19	0.25	0.27	0.32	0.21
Peak time at rt (day)	5	3	5	2	5	3
Maximum increase rate at rt (%) ^a^	105.71	64.53	145.15	36.08	77.70	32.47
Peak value at 4 °C (μg/g)	0.31	0.16	0.19	NA	0.27	ND
Peak time at 4 °C (day)	5	7	6	NA	4	ND
Maximum increase rate at 4 °C (%) ^a^	86.96	36.61	85.61	NA	46.00	ND

^a^ Maximum increase rate = (maximum content − initial content)/initial content; ^b^ Not applicable. Mango was only analyzed at room temperature since at 4 °C it could be cold-injured; ^c^ Not obtained since the contents of the compound keep rising in the storage period; ^d^ Not detectable.

**Table 2:** Initial values and increase rates of the contents of the three α-DCs in commercial juices stored separately at room temperature and 4 °C for 7 days.

	Apple	Mango	Orange	Grape	Pineapple	Peach
3-DG						
Initial value (μg/mL)	55.39	6.74	13.59	65.61	8.28	32.41
Maximum increase rate at rt (%)	60.98	116.80	38.27	8.40	108.82	86.77
Maximum increase rate at 4 °C (%)	29.63	63.50	NC ^a^	NC	63.04	43.13
GO						
Initial value (μg/mL)	0.19	0.36	0.40	0.27	0.28	0.12
Maximum increase rate at rt (%)	110.53	197.22	149.59	151.85	113.25	314.69
Maximum increase rate at 4 °C (%)	52.63	75.00	77.50	188.89	60.71	141.67
MGO						
Initial value (μg/mL)	0.56	0.51	0.25	0.28	0.28	0.83
Maximum increase rate at rt (%)	52.74	58.79	126.32	68.84	120.63	42.58
Maximum increase rate at 4 °C (%)	25.96	23.78	58.04	22.47	43.05	36.86

^a^ No significant change during storage.

**Table 3:** Initial values and increase rates of the contents of the three α-DCs in the homemade and the commercial juices stored at 4 °C for 3 days.

	Apple	Mango	Orange	Grape	Pineapple	Peach
3-DG						
Initial value (μg/mL)	4.65 (55.39) ^a^	3.09 (6.74)	1.97 (13.59)	3.60 (65.61)	4.15 (8.28)	3.52 (32.41)
Maximum increase rate at 4 °C (%)	16.72 (26.41)	45.10 (35.33)	NC (NC) ^b^	NC (NC)	18.16 (22.28)	14.20 (18.82)
GO						
Initial value ^a^ (μg/mL)	0.22 (0.19)	0.98 (0.36)	0.85 (0.40)	0.72 (0.27)	0.68 (0.28)	0.62 (0.12)
Maximum increase rate at 4 °C (%)	106.25 (23.53)	59.75 (26.23)	67.87 (57.39)	40.72 (92.09)	52.20 (38.92)	80.00 (105.22)
MGO						
Initial value (μg/mL)	ND ^c^ (0.56)	0.26 (0.51)	0.21 (0.25)	0.17 (0.28)	0.12 (0.28)	0.50 (0.83)
Maximum increase rate at 4 °C (%)	ND (15.69)	17.28 (13.58)	16.25 (22.91)	NC (NC)	33.33 (35.29)	14.00 (23.56)

^a^ Datum in bracket represents the value of the α-DC determined in commercial juices stored at 4 °C for 3 days; ^b^ No significant change during storage; ^c^ Not detectable.

## Data Availability

The original contributions presented in the study are included in the article/Supplementary Materials, further inquiries can be directed to the corresponding author.

## Supplementary Materials

The following supporting information can be downloaded at: https://www.mdpi.com/article/10.3390/foods13101509/s1, Supporting information may be found in the online version of this article.
