# Supplementary material for: Changes in α-Dicarbonyl Compound Contents during Storage of Various Fruits and Juices
Source: Foods. 2024 May 13;13(10):1509. doi: 10.3390/foods13101509 (PMC11119979; doi:10.3390/foods13101509)
Supplement: Supplementary file 1 [file foods-13-01509-s001.zip › foods-2981078-supplementary.pdf]

Supplementary Materials

Changes in α-dicarbonyl compound contents during storage of various fruits and juices

Yang Yang, Xue-Yi Liu, Qian Zhao, Dan Wu, Jin-Tao Ren, Meng Ma, Pei-Yun Li, Jia-Cai Wu, Wen-Yun Gao, and Heng Li

I. Optimization of the pretreatment process for the fruit samples:

The pretreatment procedure for the fruit samples, encompassing sampling and extraction of the α-DCs, was optimized through implementation of the experimental program outlined in Table 1. Mango and black plum were selected to represent low- and high-water-content fruits, respectively. After being peeled (only mango), cored, and sliced, they were homogenized separately. The homogenates thus obtained were weighted separately (mango 2.5 g and black plum 10 g), then one of each fruit was extracted directly with distilled water (10 mL for mango and 5 mL black plum, run 1 in Table SM1) and the other was frozen in liquid nitrogen, freeze-dried, and subsequently extracted also with distilled water (run 2 in Table SM1). After centrifugation at 11,000 rpm, 4°C for 20 min, the supernatants were transferred individually into 25 mL volumetric flasks and fixed to the volume with distilled water. In the final step, the contents of the three α-DCs in the extracts were quantified in triplicate using the procedures described sections 2.5 and 2.6, respectively. The results as depicted in Figure S1 indicate that except for 3-DG in lyophilized black plum, the contents of the analytes in both lyophilized mango and black plum samples are lower than those in the corresponding samples extracted directly. This could be attributed to the volatility of GO and MGO, which may result in their partial loss during freeze-drying. Therefore, direct water extraction was chosen to isolate the α-DCs from the fruit homogenates.

Then we tested whether a vortex- or an ultrasonic-aided method is more effective for extraction of the α-DCs. To this end, the experiments described in run 3 of Table SM1 were designed and performed. The difference between runs 1 and 3 lies in the fact that, while a vortex mixer was employed to facilitate extraction in run 1, an ultrasonic-assisted method was utilized in run 3 with all other procedures being identical. The results given in Figure S1 exhibit that in both mango and black plum, the ultrasonic-assisted method consistently yields lower extraction rates for α-DCs compared to the vortex method. Thus the vortex extraction was determined to execute the extraction of the analytes from the fruit homogenates.

In the subsequent steps, the extraction time (runs 4-6 in Table SM1) and homogenate-to-water ratio (runs 7-9 in Table SM1) were screened to optimize the extraction process. The impact of extraction time on efficiency was initially assessed. We determined that a 5-minute extraction period was necessary to achieve optimal extraction of the three analytes in both fruits. Prolonging the extraction time did not yield any additional benefits. Therefore, 5 min was chosen as the best extraction time for all fruits. Regarding the homogenate-to-water ratio, Figure S1 illustrates that a ratio of 1:4 is optimal for mango as it yields the highest concentrations of all three analytes. When black plum was extracted at a ratio of 2 : 1, the highest yields of GO and MGO were obtained, except for 3-DG whose yield at this ratio was only marginally lower than extracted at a ratio of 1 : 1. So a ratio of 2 : 1 was considered optimal for this fruit.

Table SM1 The experimental parameters for optimizing the sampling process for mango and black plum fruits.

Run	Sample	Extraction method (2800 rpm / 20kHz)	Time (min)	Homogenate-water ratio (mango/black plum)
1	FH[a]	VE[a]	5	1 : 4/2 : 1
2	LFH[a]	VE	5	1 : 4/2 : 1
3	FH	UE[a]	5	1 : 4/2 : 1
4	FH	VE	2	1 : 4/2 : 1
5	FH	VE	10	1 : 4/2 : 1
6	FH	VE	15	1 : 4/2 : 1

7	FH	VE	5	1 : 8/2: 3
8	FH	VE	5	1 : 2/1 : 1
9	FH	VE	5	1 : 1/4 : 1

[a] FH: fruit homogenate; LFH: lyophilized fruit homogenate; VE: vortex-aided extraction, UE: ultrasonic-aided extraction.

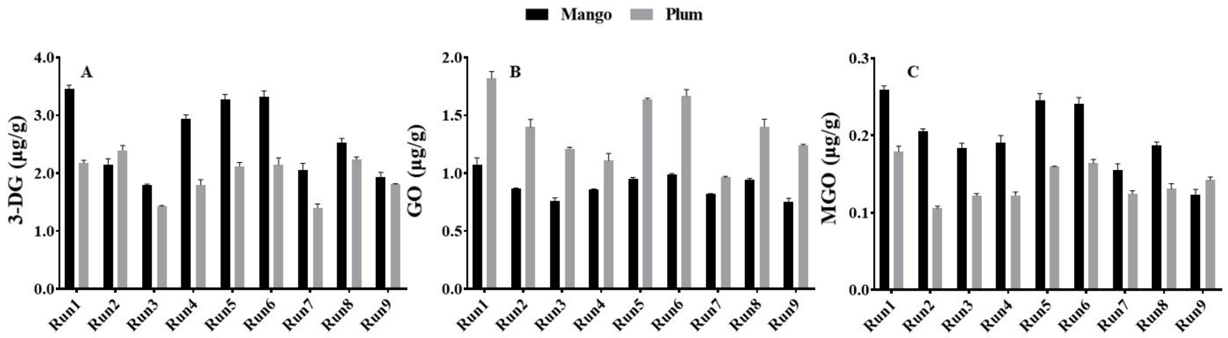

Figure S1. Optimization of the pretreatment conditions for determination of the α-DCs in mango and black plum. (A) 3-DG; (B) GO; (C) MGO (the original data are given in Supplementary Tables S3-S4).

Based on the optimization above, we decided that the preferred sampling method involves homogenizing fresh fruit and supplementing the resulting homogenate with distilled water at a homogenate-to-water ratio of 1:4 (for low-water content fruit) or 2:1 (for high-water content fruit), followed by extraction of the α-DCs using a vortex mixer operating at 2800 rpm for 5 minutes.

II. HPLC profiles of 4-nitro-1,2-phenylenediamine (NPDA) and it derivatized 3-DG, GO, and MGO:

NPDA reacted with the mixture of the three α-DCs (3-DG, GO, and MGO, 100 μM each) under the standard derivatization conditions and the products were isolated by HPLC and detected by UV and ESI-MS, respectively. The HPLC procedure was depicted in section 2.6 in

the main text and the resulting HPLC profiles are given in Figure S2. The LC-ESI-HR-MS results are listed in Table SM2. All these results show the expected products form and are completely separated by the HPLC procedure.

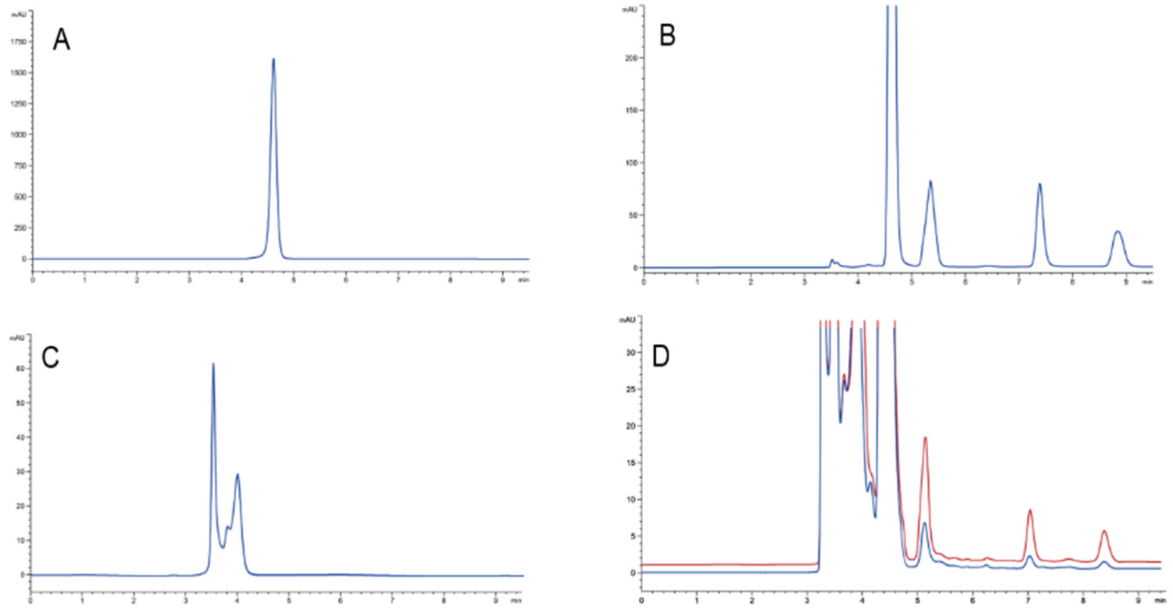

Figure S2. HPLC chromatograms obtained from (A) the blank of NPDA; (B) NPDA labeled standard 3-DG (peak 1), GO (2), and MGO (3); (C) Apricot sample without derivatization; (D) NPDA derivatized apricot sample (blue line) and apricot sample spiked with 3-DG (1.0 μg/g), GO (0.2 μg/g), and MGO (0.2 μg/g) (red line).

Table SM2 LC-ESI-HR-MS determination of NPDA-α-DC derivatives.

α-DC	Derivative	t_R (min)	Formula	*m/z* of $[M+H]^+$	
				Calcd.	Found
3-DG	NPDA-3-DG	5.40	$C_{12}H_{14}N_3O_5$	280.0933	280.0945
GO	NPDA-GO	7.44	$C_8H_5N_3O_2$	176.0460	176.0444
MGO	NPDA-MGO	8.91	$C_9H_7N_3O_2$	190.0617	190.0600

III. Validation of the HPLC procedure:

Recovery experiments were conducted to validate accuracy of the method by adding fruit and juice samples with three concentrations of the α-DC standards. For fruit samples, spiking levels were 3-DG (0.5, 1.0, and 2.0 μg/g), GO (0.1, 0.2, and 0.5 μg/g), and MGO (0.1, 0.2, and 0.5 μg/g), respectively; for juice samples, spiking levels were 3-DG (5.0, 10.0, and 25.0 μg/mL), GO (0.1, 0.2, and 0.5 μg/mL), and MGO (0.1, 0.2, and 0.5 μg/mL), respectively. After analyses with the proposed method, we found that the recoveries of all the analytes fell within the range of 85.2%-105.4% (Table SM3), indicating its suitability for determining the three α-DCs in the fruit and juice samples.

Table SM3 The recoveries of the three α-DCs in fruit and juice samples.

	Recovery (%)		
	3-DG	GO	MGO
Apricot	95.6-101.3	89.3-97.5	92.0-103.4
Plum	86.5-94.7	90.0-97.3	92.5-104.9
Nectarine	85.2-89.7	91.4-102.7	95.6-99.4
Mango	93.9-98.6	90.6-99.9	87.3-100.7
Red grapes	89.4-96.3	91.8-99.4	92.6-104.9
Orange	97.3-102.2	93.5-103.3	87.9-101.8
Apple juice	98.1-100.6	96.6-103.2	95.6-102.5
Mango juice	89.3-97.2	92.3-98.9	97.9-104.4
Grape juice	93.4-99.2	95.6-103.8	94.2-101.4
Orange juice	89.7-96.1	92.2-97.6	92.4-99.6
Pineapple juice	97.5-102.2	98.0-101.3	95.2-104.5
Peach juice	93.0-98.1	90.3-99.7	94.7-100.7

Supplementary Tables

Changes in α-dicarbonyl compound contents during storage of various fruits and juice

Yang Yang, Xue-Yi Liu, Qian Zhao, Dan Wu, Jin-Tao Ren, Meng Ma, Pei-Yun Li, Jia-Cai Wu, Wen-Yun Gao, and Heng Li

Table S1 Weight loss of fruits during storage at room temperature and 4 °C.

Storage period (days)	Weight loss (%)*			
	Apricot		Plum	
	Room temp.	4°C	Room temp.	4°C
1	0.34±0.09^{e}	0.22±0.08^{f}	0.08±0.05^{d}	0.01±0.01^{e}
2	0.61±0.17^{e}	0.28±0.07^{f}	0.11±0.04^{d}	0.02±0.01^{e}
3	2.02±0.30^{d}	0.29±0.06^{f}	0.24±0.11^{d}	0.01±0.01^{e}
4	2.51±0.56^{cd}	0.42±0.23^{ef}	0.40±0.14^{cd}	0.06±0.02^{e}
5	3.13±0.20^{c}	0.48±0.12^{ef}	1.13±0.52^{bc}	0.24±0.08^{de}
6	4.38±0.44^{b}	0.67±0.19^{def}	1.38±0.43^{b}	0.48±0.19^{cd}
7	5.59±0.53^{a}	1.00±0.33^{cdef}	1.88±0.26^{b}	0.48±0.18^{cd}
8	5.50±0.22^{a}	1.15±0.55^{cde}	2.73±1.04^{a}	0.47±0.10^{cd}
9	6.08±0.63^{a}	1.43±0.32^{cd}	2.74±0.38^{a}	0.69±0.31^{bc}
10	6.03±0.42^{a}	1.67±0.23^{bc}	2.90±0.30^{a}	0.72±0.27^{bc}
11	--	2.23±0.53^{ab}	--	1.00±0.21^{b}
12	--	2.43±0.74^{a}	--	1.91±0.17^{a}
13	--	2.44±0.90^{a}	--	1.82±0.34^{a}

Table S1 continued.

Storage period (days)	Weight loss (%)		
	Nectarine		Mango
	Room temp.	4°C	Room temp.
1	0.20±0.03^{e}	0.04±0.02^{h}	0.17±0.10^{e}
2	0.80±0.11^{e}	0.04±0.01^{h}	0.19±0.04^{e}
3	2.04±0.43^{d}	0.46±0.23^{gh}	0.24±0.05^{e}
4	3.77±0.27^{c}	0.74±0.07^{fgh}	0.29±0.06^{e}
5	4.24±0.37^{c}	1.13±0.34^{fg}	1.71±0.05^{d}
6	5.58±1.43^{b}	1.34±0.31^{ef}	3.71±0.31^{c}
7	6.38±0.46^{ab}	1.30±0.43^{ef}	4.98±1.04^{b}
8	6.33±0.51^{ab}	1.91±0.68^{de}	7.46±0.36^{a}
9	6.83±1.24^{ab}	2.14±0.63^{cd}	8.07±0.82^{a}
10	7.05±0.88^{a}	2.61±0.25^{bc}	8.52±1.19^{a}
11	--	2.70±0.23^{bc}	8.39±1.08^{a}
12	--	3.01±0.59^{ab}	8.19±0.34^{a}
13	--	3.40±0.45^{a}	--

Table S1 continued.

Storage period (days)	Weight loss (%)			
	Orange		Grape	
	Room temp.	4°C	Room temp.	4°C
1	0.01±0.01^{e}	0.01±0.01^{d}	0.36±0.17^{e}	0.30±0.16^{f}
2	0.02±0.01^{e}	0.03±0.02^{d}	0.88±0.12^{de}	0.88±0.13^{e}
3	0.23±0.18^{de}	0.3±0.12^{d}	1.89±0.36^{d}	1.60±0.28^{d}
4	0.64±0.21^{de}	0.79±0.20^{cd}	3.15±0.52^{c}	1.83±0.46^{d}
5	1.41±0.42^{cd}	1.41±0.34^{bc}	3.58±0.96^{c}	2.48±0.28^{c}
6	2.32±1.15^{bc}	1.90±0.67^{ab}	5.06±0.23^{b}	2.59±0.24^{bc}
7	2.92±0.72^{b}	2.56±0.72^{a}	6.62±0.94^{a}	3.05±0.10^{ab}
8	4.43±1.37^{a}	2.65±0.73^{a}	7.15±0.76^{a}	3.52±0.35^{a}

*Mean values in the same column with different letters are significantly different at 5% confidence level.

Table S2 Optimization of the derivatization conditions for the reactions between NPDA and the three α-DCs.

	3-DG (mAU)	GO (mAU)	MGO (mAU)
1*	18.23±1.29	13.06±0.46	3.72±0.24
2	49.28±0.91	35.74±0.38	9.61±0.31
3	111.91±1.26	59.82±0.39	34.44±0.63
4	172.87±2.05	89.34±0.45	59.93±1.28
5	172.94±1.19	91.08±0.82	66.58±0.52
6	5.57±0.31	15.63±0.38	65.57±1.96
7	6.97±0.33	16.50±0.43	72.63±1.11
8	10.53±0.45	17.97±1.19	77.33±4.46
9	11.13±0.54	17.83±0.21	73.90±1.14
10	9.43±0.21	1 7.07±0.25	14.90±0.65
11	122.10±2.48	89.27±2.29	55.33±0.68
12	124.47±2.01	99.33±1.51	56.40±0.29
13	121.50±6.20	72.03±2.53	42.23±5.35
14	81.43±1.50	52.22±1.38	18.31±0.29
15	113.10±2.26	72.79±2.07	31.63±0.98
16	136.20±2.66	88.25±0.70	40.52±1.00
17	160.92±1.56	106.5±0.47	47.94±0.73
18	175.54±1.21	116.02±1.33	56.36±1.13
19	174.89±8.50	112.5±1.55	50.92±1.68
20	176.53±0.78	108.00±0.00	68.13±0.63
21	106.67±0.95	97.93±0.21	58.67±0.26
22	68.07±2.22	88.37±0.24	53.63±1.37
23	35.60±1.02	67.00±2.41	54.97±0.84

*Lines 1-5: the ratio of NPDA to total α-DCs; 6-13: the acidity of the reaction system; 14-19: the reaction time; 20-23: the reaction temperature.

Table S3 Optimization of the pretreatment conditions for determination of the α-DCs in mango

	Run1	Run2	Run3	Run4	Run5
3-DG (μg/g)	3.45±0.06	2.15±0.08	1.79±0.02	2.94±0.05	3.27±0.07
GO (μg/g)	1.08±0.05	0.87±0.00	0.76±0.02	0.86±0.01	0.95±0.01
MGO (μg/g)	0.26±0.00	0.21±0.00	0.18±0.01	0.19±0.01	0.25±0.01

Table S3 continued.

	Run6	Run7	Run8	Run9
3-DG (μg/g)	3.32±0.08	2.06±0.09	2.53±0.06	1.93±0.07
GO (μg/g)	0.99±0.01	0.82±0.00	0.94±0.01	0.75±0.03
MGO (μg/g)	0.24±0.01	0.15±0.01	0.19±0.00	0.12±0.01

mean ± SD, n=3.

Table S4 Optimization of the pretreatment conditions for determination of the α-DCs in plum

	Run1	Run2	Run3	Run4	Run5
3-DG (μg/g)	2.18±0.04	2.39±0.07	1.43±0.01	1.80±0.07	2.12±0.05
GO (μg/g)	1.82±0.05	1.40±0.05	1.21±0.01	1.11±0.05	1.64±0.01
MGO (μg/g)	0.18±0.01	0.11±0.00	0.12±0.00	0.12±0.00	0.16±0.00

Table S4 continued.

	Run6	Run7	Run8	Run9
3-DG (μg/g)	2.14±0.10	1.40±0.05	2.07±0.03	1.81±0.01
GO (μg/g)	1.67±0.04	0.96±0.01	1.40±0.05	1.24±0.01
MGO (μg/g)	0.16±0.00	0.12±0.00	0.13±0.01	0.14±0.00

mean ± SD, n=3.

Tables S5 3-DG, GO, and MGO concentrations in apricots during storage at room temperature.

Storage time (day)	3-DG (μg/g)	GO (μg/g)	MGO (μg/g)
0	1.58±0.13^{f}	0.65±0.02^{c}	0.16±0.01^{d}
1	1.88±0.22^{ef}	0.83±0.02^{bc}	0.25±0.01^{b}
2	2.66±0.26^{ab}	1.18±0.03^{a}	0.28±0.01^{b}
3	2.35±0.09^{abcd}	1.31±0.06^{a}	0.28±0.02^{b}
4	2.73±0.20^{a}	1.17±0.10^{a}	0.34±0.03^{a}

5	2.64±0.08^{ab}	0.91±0.05^{b}	0.34±0.01^{a}
6	2.30±0.17^{bcd}	0.85±0.03^{b}	0.28±0.01^{b}
7	2.45±0.30^{abc}	0.86±0.10^{b}	0.26±0.02^{b}
8	2.16±0.12^{cde}	0.73±0.03^{bc}	0.24±0.03^{bc}
9	1.96±0.10^{def}	0.77±0.07^{bc}	0.23±0.01^{bc}
10	1.79±0.12^{ef}	0.74±0.08^{bc}	0.20±0.04^{cd}

mean ± SD, n=3. Mean values in the same column with different letters are significantly different at 5% confidence level.

Table S6 3-DG, GO, and MGO concentrations in apricots during storage at 4°C.

Storage time (day)	3-DG (μg/g)	GO (μg/g)	MGO (μg/g)
0	1.58±0.13^{de}	0.65±0.02^{de}	0.16±0.01^{gh}
1	1.52±0.13^{de}	0.79±0.06^{bc}	0.23±0.01^{cde}
2	1.65±0.11^{cde}	0.89±0.02^{b}	0.23±0.03^{cde}
3	1.54±0.28^{de}	0.84±0.02^{b}	0.28±0.01^{ab}
4	1.83±0.14^{bcd}	1.03±0.12^{a}	0.25±0.02^{bcd}
5	1.75±0.21^{bcd}	1.11±0.05^{a}	0.31±0.03^{a}
6	1.90±0.08^{bcd}	0.81±0.03^{b}	0.25±0.01^{bc}
7	2.08±0.34^{abc}	0.77±0.04^{bcd}	0.23±0.00^{cde}
8	2.12±0.08^{ab}	0.65±0.03^{de}	0.21±0.01^{def}
9	2.44±0.20^{a}	0.67±0.11^{cde}	0.21±0.01^{ef}
10	2.40±0.07^{a}	0.65±0.03^{de}	0.19±0.01^{fg}
11	2.12±0.07^{ab}	0.56±0.08^{ef}	0.16±0.01^{gh}
12	1.52±0.20^{de}	0.45±0.04^{f}	0.15±0.01^{h}
13	1.27±0.31^{e}	0.46±0.02^{f}	0.14±0.02^{h}

mean ± SD, n=3.

Table S7 3-DG, GO, and MGO concentrations in plums during storage at room temperature.

Storage time (day)	3-DG (μg/g)	GO (μg/g)	MGO (μg/g)
0	$1.64±0.32^{c}$	$1.16±0.09^{e}$	$0.11±0.01^{f}$
1	$2.13±0.06^{ab}$	$1.21±0.18^{de}$	$0.15±0.01^{bc}$
2	$1.96±0.11^{ab}$	$1.31±0.03^{cde}$	$0.15±0.01^{cd}$
3	$2.42±0.17^{a}$	$1.30±0.07^{cde}$	$0.19±0.01^{a}$
4	$2.35±0.28^{ab}$	$1.48±0.18^{bc}$	$0.18±0.01^{ab}$
5	$2.49±0.10^{a}$	$1.84±0.06^{a}$	$0.16±0.01^{bc}$
6	$2.33±0.11^{ab}$	$1.43±0.09^{bcd}$	$0.15±0.01^{cd}$
7	$2.14±0.10^{ab}$	$1.57±0.09^{b}$	$0.14±0.02^{cde}$
8	$1.96±0.25^{bc}$	$1.27±0.04^{cde}$	$0.13±0.01^{def}$
9	$1.67±0.12^{c}$	$1.15±0.12^{e}$	$0.12±0.01^{ef}$
10	$1.22±0.05^{d}$	$1.09±0.05^{e}$	$0.12±0.01^{f}$

mean ± SD, n=3.

Table S8 3-DG, GO, and MGO concentrations in plums during storage at 4°C.

Storage time (day)	3-DG (μg/g)	GO (μg/g)	MGO (μg/g)
0	$1.64±0.32^{cd}$	$1.16±0.09^{a}$	$0.11±0.01^{ef}$
1	$1.66±0.10^{cd}$	$1.15±0.04^{a}$	$0.12±0.01^{def}$
2	$1.82±0.14^{bcd}$	$1.11±0.09^{a}$	$0.12±0.00^{def}$
3	$2.09±0.09^{ab}$	$1.07±0.05^{a}$	$0.13±0.02^{abcde}$
4	$1.93±0.06^{abc}$	$1.06±0.16^{a}$	$0.13±0.00^{abcde}$
5	$2.14±0.22^{ab}$	$1.19±0.08^{a}$	$0.15±0.01^{abc}$
6	$2.20±0.15^{a}$	$1.18±0.06^{a}$	$0.14±0.01^{abcd}$
7	$1.90±0.10^{abc}$	$1.15±0.10^{a}$	$0.16±0.00^{a}$
8	$1.51±0.15^{de}$	$1.08±0.06^{a}$	$0.15±0.02^{ab}$
9	$1.66±0.08^{cd}$	$1.03±0.09^{a}$	$0.14±0.02^{abcde}$

10	1.30±0.09[ef]	1.00±0.01[a]	0.13±0.01[bcde]
11	1.18±0.05[f]	1.11±0.05[a]	0.12±0.01[cdef]
12	1.06±0.17[f]	0.99±0.09[a]	0.12±0.01[ef]
13	0.99±0.07[f]	1.16±0.16[a]	0.10±0.00[f]

mean ± SD, n=3.

Table S9 3-DG, GO, and MGO concentrations in nectarines during storage at room temperature.

Storage time (day)	3-DG (μg/g)	GO (μg/g)	MGO (μg/g)
0	1.65±0.10[fg]	0.45±0.05[f]	0.10±0.01[f]
1	1.93±0.09[ef]	0.52±0.07[f]	0.14±0.02[de]
2	2.32±0.15[bc]	1.30±0.04[c]	0.16±0.01[bcde]
3	2.50±0.08[bc]	1.23±0.05[c]	0.15±0.01[de]
4	2.82±0.07[a]	1.85±0.13[b]	0.19±0.03[bc]
5	2.43±0.09[bc]	2.32±0.07[a]	0.25±0.02[a]
6	2.62±0.23[ab]	1.01±0.03[d]	0.17±0.01[bcd]
7	2.31±0.16[bc]	0.84±0.05[e]	0.19±0.02[b]
8	2.23±0.22[cd]	0.63±0.02[f]	0.15±0.01[cde]
9	1.97±0.09[de]	0.55±0.04[f]	0.16±0.01[cde]
10	1.61±0.09[g]	0.48±0.08[f]	0.13±0.02[e]

mean ± SD, n=3.

Table S10 3-DG, GO, and MGO concentrations in nectarines during storage at 4℃.

Storage time (day)	3-DG (μg/g)	GO (μg/g)	MGO (μg/g)
0	1.65±0.101[f]	0.45±0.05[a]	0.10±0.01[d]
1	1.67±0.072[ef]	0.40±0.05[a]	0.10±0.02[d]
2	1.71±0.056[ef]	0.49±0.07[a]	0.12±0.01[cd]
3	1.73±0.185[ef]	0.47±0.02[a]	0.15±0.01[bc]
4	1.92±0.100[cde]	0.48±0.11[a]	0.17±0.01[ab]

5	1.84±0.020^{def}	0.51±0.04^{a}	0.15±0.02^{b}
6	2.11±0.023^{abc}	0.41±0.04^{a}	0.19±0.02^{a}
7	2.04±0.043^{bcd}	0.43±0.02^{a}	0.14±0.01^{bcd}
8	2.20±0.133^{ab}	0.41±0.02^{a}	0.11±0.00^{cd}
9	2.30±0.165^{a}	0.39±0.01^{a}	0.10±0.01^{d}
10	2.15±0.094^{abc}	0.36±0.01^{a}	0.10±0.01^{d}
11	1.65±0.102^{f}	0.38±0.02^{a}	0.12±0.01^{d}
12	1.42±0.074^{g}	0.38±0.01^{a}	0.10±0.02^{d}
13	1.07±0.130^{h}	0.47±0.07^{a}	0.10±0.01^{d}

mean ± SD, n=3.

Table S11 3-DG, GO, and MGO concentrations in mangoes during storage at room temperature.

Storage time (day)	3-DG (μg/g)	GO (μg/g)	MGO (μg/g)
0	2.45±0.06^{d}	0.81±0.07d^{ef}	0.20±0.01^{bc}
1	2.30±0.12^{d}	0.77±0.05^{ef}	0.23±0.03^{b}
2	2.45±0.15^{d}	0.84±0.03^{def}	0.27±0.02^{a}
3	2.62±0.10^{d}	0.77±0.07^{ef}	0.26±0.04^{a}
4	2.30±0.13^{d}	0.92±0.03^{cd}	0.19±0.03^{cd}
5	2.58±0.36^{d}	1.05±0.11^{b}	0.17±0.01^{cde}
6	2.73±0.11^{cd}	1.01±0.04^{bc}	0.14±0.01^{efg}
7	2.73±0.14^{cd}	1.08±0.04^{b}	0.14±0.01^{efg}
8	3.10±0.16^{bc}	1.23±0.05^{a}	0.14±0.00^{efg}
9	3.77±0.08^{a}	0.91±0.04^{cd}	0.16±0.02^{def}
10	3.46±0.29^{ab}	0.88±0.02^{de}	0.13±0.01^{fg}
11	3.21±0.38^{b}	0.86±0.05^{def}	0.12±0.01^{g}
12	2.64±0.10^{d}	0.73±0.08^{f}	0.11±0.00^{g}

mean ± SD, n=3.

Table S12 3-DG, GO, and MGO concentrations in sugar oranges during storage at room temperature.

Storage time (day)	3-DG (μg/g)	GO (μg/g)	MGO (μg/g)
0	1.78±0.07^{f}	0.77±0.02^{f}	0.18±0.01^{e}
1	3.13±0.32^{e}	1.67±0.17^{d}	0.24±0.02^{cd}
2	4.03±0.21^{de}	1.38±0.06^{e}	0.19±0.01^{de}
3	7.32±0.30^{b}	2.43±0.13^{c}	0.27±0.01^{bc}
4	8.46±1.02^{a}	3.23±0.10^{a}	0.30±0.01^{ab}
5	5.79±0.29^{b}	2.82±0.06^{b}	0.32±0.05^{a}
6	5.05±0.20^{cd}	3.12±0.15^{a}	0.29±0.02^{ab}
7	4.06±0.23^{de}	2.36±0.08^{c}	0.27±0.02^{bc}
8	3.71±0.37^{e}	1.87±0.09^{d}	0.25±0.01^{bc}

mean ± SD, n=3.

Table S13 3-DG, GO, and MGO concentrations in sugar oranges during storage at 4°C.

Storage time (day)	3-DG (μg/g)	GO (μg/g)	MGO (μg/g)
su0	1.78±0.07^{e}	0.77±0.02^{d}	0.18±0.01^{b}
1	4.83±0.35^{d}	0.96±0.08^{cd}	0.19±0.01^{b}
2	6.94±0.31^{c}	1.12±0.04^{c}	0.19±0.01^{b}
3	7.78±0.31^{c}	1.84±0.15^{b}	0.19±0.01^{b}
4	10.00±0.32^{a}	2.97±0.14^{a}	0.27±0.02^{a}
5	8.90±0.19^{b}	2.01±0.07^{b}	0.26±0.01^{a}
6	8.86±0.95^{b}	0.92±0.02^{cd}	0.24±0.01^{a}
7	7.60±0.33^{c}	0.89±0.13^{d}	0.21±0.03^{b}
8	7.57±0.13^{c}	0.83±0.02^{d}	0.20±0.01^{b}

mean ± SD, n=3.

Table S14 3-DG, GO, and MGO concentrations in red grapes during storage at room temperature.

Storage time (day)	3-DG (μg/g)	GO (μg/g)	MGO (μg/g)
0	3.33±0.35^{d}	0.66±0.00^{d}	0.16±0.01^{c}
1	4.35±0.28^{c}	0.68±0.02^{cd}	0.17±0.01^{bc}
2	4.94±0.19^{bc}	0.67±0.03^{d}	0.21±0.01^{a}
3	4.64±0.33^{bc}	0.70±0.01^{cd}	0.21±0.01^{a}
4	6.06±0.13^{a}	0.74±0.02^{bc}	0.20±0.01^{ab}
5	5.39±0.38^{ab}	0.79±0.04^{ab}	0.19±0.01^{abc}
6	5.09±0.18^{bc}	0.78±0.04^{ab}	0.19±0.03^{abc}
7	5.03±0.66^{bc}	0.79±0.03^{a}	0.18±0.01^{bc}
8	4.42±0.34^{c}	0.81±0.03^{a}	0.17±0.01^{bc}

mean ± SD, n=3.

Table S15 3-DG, GO, and MGO concentrations in red grapes during storage at 4°C.

Storage time (day)	3-DG (μg/g)	GO (μg/g)	MGO (μg/g)
0	3.33±0.35^{e}	0.66±0.00^{c}	0.16±0.02^{a}
1	4.57±0.19^{d}	0.66±0.02^{c}	0.15±0.02^{a}
2	5.13±0.22^{d}	0.67±0.04^{bc}	0.18±0.01^{a}
3	6.23±0.66^{c}	0.66±0.01^{c}	0.18±0.00^{a}
4	6.45±0.25^{abc}	0.72±0.01^{ab}	0.19±0.02^{a}
5	6.30±0.26^{bc}	0.74±0.02^{a}	0.19±0.01^{a}
6	7.01±0.07^{ab}	0.71±0.03^{abc}	0.18±0.03^{a}
7	7.11±0.45^{a}	0.68±0.01^{bc}	0.16±0.00^{a}
8	7.23±0.13^{a}	0.66±0.02^{c}	0.17±0.01^{a}

mean ± SD, n=3.

Table S16 3-DG, GO, and MGO concentrations in commercial apple juice during storage at room temperature.

Storage time (day)	3-DG (μg/mL)	GO (μg/mL)	MGO (μg/mL)
0	55.39±1.58^{e}	0.19±0.01^{d}	0.56±0.01^{f}
1	64.88±2.58^{d}	0.25±0.02^{cd}	0.68±0.02^{de}
2	76.74±1.09^{bc}	0.22±0.02^{cd}	0.64±0.02^{e}
3	72.69±1.81^{c}	0.36±0.01^{ab}	0.74±0.02^{bcd}
4	83.54±4.18^{ab}	0.30±0.03^{bc}	0.70±0.05^{cde}
5	79.22±2.66^{bc}	0.36±0.02^{ab}	0.77±0.03^{bc}
6	86.52±3.55^{a}	0.39±0.05^{ab}	0.80±0.02^{ab}
7	89.17±2.64^{a}	0.40±0.03^{a}	0.86±0.04^{a}

mean ± SD, n=3.

Table S17 3-DG, GO, and MGO concentrations in commercial apple juice during storage at 4°C.

Storage time (day)	3-DG (μg/mL)	GO (μg/mL)	MGO (μg/mL)
0	55.39±1.58^{b}	0.19±0.01^{bc}	0.56±0.01^{c}
1	62.04±3.80^{a}	0.17±0.02^{c}	0.54±0.02^{c}
2	64.33±1.15^{a}	0.19±0.04^{bc}	0.60±0.03^{bc}
3	70.02±5.00^{a}	0.24±0.04^{ab}	0.65±0.01^{ab}
4	64.61±2.65^{a}	0.24±0.04^{ab}	0.63±0.04^{ab}
5	70.43±2.24^{a}	0.27±0.01^{ab}	0.67±0.02^{ab}
6	71.37±3.27^{a}	0.28±0.02^{a}	0.70±0.03^{a}
7	71.80±1.77^{a}	0.29±0.04^{a}	0.71±0.03^{a}

mean ± SD, n=3.

Table S18 3-DG, GO, and MGO concentrations in commercial mango juice during storage at room temperature.

Storage time (day)	3-DG (μg/mL)	GO (μg/mL)	MGO (μg/mL)
0	6.74±0.69^{d}	0.36±0.01^{d}	0.51±0.02^{e}
1	9.60±0.66^{c}	0.58±0.05^{d}	0.55±0.03^{e}
2	10.60±0.88^{bc}	0.52±0.05^{d}	0.68±0.01^{bcd}
3	10.07±0.11^{c}	0.70±0.04^{c}	0.60±0.02^{de}
4	11.60±0.77^{bc}	0.85±0.01^{b}	0.68±0.06^{cd}
5	11.07±0.51^{bc}	0.74±0.04^{c}	0.75±0.02^{abc}
6	12.40±1.00^{b}	1.00±0.02^{a}	0.79±0.01^{ab}
7	14.61±0.47^{a}	1.07±0.02^{a}	0.82±0.05^{a}

mean ± SD, n=3.

Table S19 3-DG, GO, and MGO concentrations in commercial mango juice during storage at 4℃.

Storage time (day)	3-DG (μg/mL)	GO (μg/mL)	MGO (μg/mL)
0	6.74±0.69^{c}	0.36±0.01^{d}	0.51±0.01^{b}
1	7.68±0.82^{c}	0.37±0.03^{d}	0.53±0.04^{b}
2	7.12±0.34^{c}	0.46±0.02^{c}	0.52±0.06^{b}
3	9.12±1.48^{abc}	0.44±0.04^{c}	0.58±0.03^{ab}
4	7.93±0.71^{bc}	0.57±0.02^{ab}	0.58±0.02^{ab}
5	9.44±0.60^{abc}	0.54±0.02^{b}	0.62±0.03^{ab}
6	10.41±1.18^{ab}	0.62±0.04^{a}	0.64±0.01^{a}
7	11.02±0.71^{a}	0.63±0.04^{a}	0.64±0.02^{a}

mean ± SD, n=3.

Table S20 3-DG, GO, and MGO concentrations in commercial orange juice during storage at room temperature.

Storage time (day)	3-DG (μg/mL)	GO (μg/mL)	MGO (μg/mL)
0	13.59±0.80^{c}	0.40±0.02^{d}	0.25±0.03^{f}
1	14.17±1.61^{bc}	0.53±0.01^{c}	0.29±0.01^{ef}
2	14.83±0.15^{bc}	0.60±0.02^{c}	0.37±0.01^{de}
3	14.40±0.61^{bc}	0.74±0.05^{b}	0.49±0.03^{bc}
4	15.06±1.15^{bc}	0.96±0.02^{a}	0.43±0.03^{cd}
5	16.58±1.63^{abc}	0.89±0.04^{a}	0.52±0.01^{ab}
6	17.56±0.61^{ab}	0.98±0.03^{a}	0.54±0.02^{ab}
7	18.79±1.33^{a}	1.00±0.08^{a}	0.57±0.02^{a}

mean ± SD, n=3.

STable 21 3-DG, GO, and MGO concentration in commercial orange juice during storage at 4°C.

Storage time (day)	3-DG (μg/mL)	GO (μg/mL)	MGO (μg/mL)
0	13.59±0.80^{a}	0.40±0.02^{b}	0.25±0.03^{c}
1	12.81±0.62^{a}	0.42±0.03^{b}	0.29±0.02^{bc}
2	13.32±1.02^{a}	0.40±0.02^{b}	0.29±0.02^{bc}
3	13.19±1.39^{a}	0.63±0.02^{a}	0.31±0.01^{abc}
4	13.78±0.88^{a}	0.64±0.04^{a}	0.36±0.01^{ab}
5	13.88±0.41^{a}	0.65±0.01^{a}	0.34±0.05^{abc}
6	14.09±0.68^{a}	0.64±0.03^{a}	0.39±0.01^{ab}
7	12.92±0.87^{a}	0.71±0.03^{a}	0.40±0.06^{a}

mean ± SD, n=3.

Table S22 3-DG, GO, and MGO concentrations in commercial grape juice during storage at room temperature.

Storage time (day)	3-DG (μg/mL)	GO (μg/mL)	MGO (μg/mL)
0	65.61 ± 1.53^{c}	0.27 ± 0.01^{f}	0.28 ± 0.01^{c}
1	65.05 ± 1.10^{c}	0.33 ± 0.02^{e}	0.29 ± 0.02^{c}
2	67.45 ± 1.07^{ab}	0.39 ± 0.02^{d}	0.33 ± 0.05^{ab}
3	67.46 ± 2.59^{ab}	0.49 ± 0.02^{c}	0.35 ± 0.03^{ab}
4	68.52 ± 0.72^{ab}	0.49 ± 0.04^{c}	0.34 ± 0.02^{ab}
5	68.15 ± 1.42^{ab}	0.56 ± 0.01^{b}	0.37 ± 0.02^{ab}
6	69.16 ± 0.82^{ab}	0.59 ± 0.01^{b}	0.39 ± 0.01^{b}
7	71.12 ± 0.91^{a}	0.68 ± 0.04^{a}	0.48 ± 0.04^{a}

mean ± SD, n=3.

Table S23 3-DG, GO, and MGO concentrations in commercial grape juice during storage at 4℃.

Storage time (day)	3-DG (μg/mL)	GO (μg/mL)	MGO (μg/mL)
0	65.61 ± 1.53^{a}	0.27 ± 0.01^{f}	0.28 ± 0.01^{b}
1	64.46 ± 0.49^{a}	0.36 ± 0.03^{e}	0.28 ± 0.02^{b}
2	64.05 ± 1.31^{a}	0.51 ± 0.01^{cd}	0.29 ± 0.01^{b}
3	63.99 ± 0.40^{a}	0.45 ± 0.03^{d}	0.28 ± 0.01^{b}
4	64.75 ± 1.01^{a}	0.55 ± 0.04^{c}	0.30 ± 0.02^{b}
5	63.21 ± 0.62^{a}	0.64 ± 0.03^{b}	0.31 ± 0.01^{b}
6	64.05 ± 1.67^{a}	0.70 ± 0.03^{b}	0.32 ± 0.02^{ab}
7	64.44 ± 1.13^{a}	0.78 ± 0.03^{a}	0.35 ± 0.01^{a}

mean ± SD, n=3.

Table S24 3-DG, GO, and MGO concentrations in commercial pineapple juice during storage at room temperature.

Storage time (day)	3-DG (μg/mL)	GO (μg/mL)	MGO (μg/mL)
0	8.28±0.16^{e}	0.28±0.02^{e}	0.28±0.02^{c}
1	11.93±0.65^{cd}	0.34±0.01^{d}	0.32±0.01^{c}
2	9.84±1.17^{de}	0.42±0.05^{c}	0.42±0.02^{b}
3	12.72±0.74^{c}	0.47±0.02^{bc}	0.43±0.05^{b}
4	14.72±1.33^{bc}	0.57±0.01^{a}	0.57±0.02^{a}
5	13.71±1.70^{abc}	0.52±0.03^{ab}	0.58±0.01^{a}
6	16.26±0.98^{ab}	0.59±0.02^{a}	0.59±0.04^{a}
7	17.29±1.17^{a}	0.60±0.04^{a}	0.62±0.02^{a}

mean ± SD, n=3.

Table S25 3-DG, GO, and MGO concentrations in commercial pineapple juice during storage at 4℃.

Storage time (day)	3-DG (μg/mL)	GO (μg/mL)	MGO (μg/mL)
0	8.28±0.16^{c}	0.28±0.02^{b}	0.28±0.02^{c}
1	8.07±0.77^{c}	0.28±0.01^{b}	0.29±0.02^{c}
2	7.74±0.59^{c}	0.33±0.01^{b}	0.33±0.03^{bc}
3	10.12±0.90^{c}	0.39±0.02^{a}	0.38±0.01^{ab}
4	9.09±0.72^{c}	0.42±0.05^{a}	0.43±0.01^{a}
5	11.81±1.32^{ab}	0.41±0.01^{a}	0.40±0.04^{ab}
6	12.90±1.22^{a}	0.40±0.02^{a}	0.42±0.03^{a}
7	13.50±0.86^{a}	0.45±0.01^{a}	0.40±0.03^{ab}

mean ± SD, n=3.

Table S26 3-DG, GO, and MGO concentrations in commercial peach juice during storage at room temperature.

Storage time (day)	3-DG (μg/mL)	GO (μg/mL)	MGO (μg/mL)
0	32.41 ± 1.22^{g}	0.12 ± 0.02^{e}	0.83 ± 0.02^{c}
1	36.62 ± 2.75^{f}	0.17 ± 0.01^{e}	0.90 ± 0.06^{bc}
2	45.75 ± 0.87^{f}	0.29 ± 0.03^{d}	0.92 ± 0.01^{bc}
3	41.30 ± 2.00^{e}	0.34 ± 0.02^{c}	0.97 ± 0.01^{b}
4	52.42 ± 1.32^{c}	0.43 ± 0.01^{b}	1.11 ± 0.05^{a}
5	50.43 ± 1.79^{c}	0.37 ± 0.01^{c}	1.14 ± 0.01^{a}
6	56.61 ± 1.08^{b}	0.47 ± 0.03^{ab}	1.10 ± 0.01^{a}
7	60.54 ± 1.96^{a}	0.50 ± 0.02^{a}	1.18 ± 0.07^{a}

mean ± SD, n=3.

Table S27 3-DG, GO, and MGO concentrations in commercial peach juice during storage at 4°C.

Storage time (day)	3-DG (μg/mL)	GO (μg/mL)	MGO (μg/mL)
0	32.41 ± 1.22^{d}	0.12 ± 0.02^{c}	0.83 ± 0.02^{c}
1	35.27 ± 1.80^{cd}	0.12 ± 0.02^{c}	0.89 ± 0.03^{c}
2	34.06 ± 1.56^{d}	0.20 ± 0.02^{b}	0.87 ± 0.01^{c}
3	38.51 ± 1.17^{c}	0.25 ± 0.00^{ab}	1.02 ± 0.01^{b}
4	35.08 ± 2.51^{cd}	0.24 ± 0.01^{ab}	1.07 ± 0.01^{ab}
5	42.43 ± 1.13^{b}	0.25 ± 0.01^{ab}	1.07 ± 0.06^{ab}
6	43.74 ± 1.34^{ab}	0.27 ± 0.04^{ab}	1.10 ± 0.01^{ab}
7	46.39 ± 0.96^{a}	0.29 ± 0.05^{a}	1.13 ± 0.03^{a}

mean ± SD, n=3.

Table S28 3-DG, GO, and MGO concentrations in home-made apple juice during storage at 4°C.

Storage time (day)	3-DG (μg/g)	GO (μg/g)	MGO (μg/g)
0	4.65±0.04[c]	0.22±0.01[d]	ND
1	5.10±0.08[b]	0.26±0.01[c]	ND
2	5.42±0.10[a]	0.39±0.03[b]	ND
3	5.41±0.16[a]	0.46±0.00[a]	ND

mean ± SD, n=3. ND: no detection

Table S29 3-DG, GO, and MGO concentrations in home-made mango juice during storage at 4°C.

Storage time (day)	3-DG (μg/g)	GO (μg/g)	MGO (μg/g)
0	3.09±0.02[c]	0.98±0.12[b]	0.26±0.00[b]
1	3.23±0.13[c]	0.99±0.08[b]	0.27±0.03[ab]
2	4.15±0.13[b]	1.34±0.17[a]	0.30±0.01[ab]
3	4.49±0.20[a]	1.56±0.12[a]	0.31±0.02[a]

mean ± SD, n=3.

Table S30 3-DG, GO, and MGO concentrations in home-made orange juice during storage at 4°C.

Storage time (day)	3-DG (μg/g)	GO (μg/g)	MGO (μg/g)
0	1.97±0.14[a]	0.85±0.04[c]	0.21±0.00[b]
1	1.87±0.07[a]	1.15±0.03[b]	0.21±0.01[b]
2	2.00±0.13[a]	1.42±0.10[a]	0.22±0.01[b]
3	1.97±0.08[a]	1.41±0.16[a]	0.24±0.01[a]

mean ± SD, n=3.

Table S31 3-DG, GO, and MGO concentrations in home-made grape juice during storage at 4℃.

Storage time (day)	3-DG (μg/g)	GO (μg/g)	MGO (μg/g)
0	3.60±0.22^{a}	0.72±0.02^{c}	0.17±0.01^{a}
1	3.66±0.08^{a}	0.92±0.03^{b}	0.18±0.01^{a}
2	3.71±0.14^{a}	0.97±0.01^{a}	0.18±0.00^{a}
3	3.61±0.06^{a}	1.01±0.01^{a}	0.17±0.01^{a}

mean ± SD, n=3.

Table S32 3-DG, GO, and MGO concentrations in home-made pineapple juice during storage at 4℃.

Storage time (day)	3-DG (μg/g)	GO (μg/g)	MGO (μg/g)
0	4.15±0.13^{c}	0.68±0.02^{c}	0.12±0.01^{b}
1	4.45±0.20^{bc}	0.89±0.01^{b}	0.15±0.01^{a}
2	4.55±0.17^{b}	0.93±0.06^{b}	0.16±0.01^{a}
3	4.90±0.02^{a}	1.04±0.04^{a}	0.16±0.01^{a}

mean ± SD, n=3.

Table S33 3-DG, GO, and MGO concentrations in home-made peach juice during storage at 4℃.

Storage time (day)	3-DG (μg/g)	GO (μg/g)	MGO (μg/g)
0	3.52±0.15^{b}	0.62±0.00^{d}	0.50±0.01^{b}
1	3.67±0.11^{b}	0.75±0.01^{c}	0.50±0.01^{b}
2	3.98±0.12^{a}	1.05±0.05^{b}	0.54±0.02^{a}
3	4.02±0.09^{a}	1.12±0.03^{a}	0.57±0.01^{a}

mean ± SD, n=3.
